# Design of Nano Screw Pump for Water Transport and its Mechanisms

**DOI:** 10.1038/srep41717

**Published:** 2017-02-03

**Authors:** LiYa Wang, HengAn Wu, FengChao Wang

**Affiliations:** 1 CAS Key Laboratory of Mechanical Behavior and Design of Materials, Department of Modern Mechanics, CAS Center for Excellence in Nanoscience, University of Science and Technology of China, Hefei, Anhui 230027, China

## Abstract

Nanopumps conducting fluids through nanochannels have attracted considerable interest for their potential applications in nanofiltration, water desalination and drug delivery. Here, we demonstrate by molecular dynamics (MD) simulations that a nano screw pump is designed with helical nanowires embedded in a nanochannel, which can be used to drive unidirectional water flow. Such helical nanowires have been successfully synthesized in many experiments. By investigating the water transport mechanism through nano screw pumps with different configuration parameters, three transport modes were observed: cluster-by-cluster, pseudo-continuous, and linear-continuous, in which the water flux increases linearly with the rotating speed. The influences of the nanowires’ surface energy and the screw’s diameter on water transport were also investigated. Results showed that the water flux rate increases as the decreasing wettability of helical nanowires. The deviation in water flux in screw pumps with smaller radius is attributed to the weak hydrogen bonding due to space confinement and the hydrophobic blade. Moreover, we also proposed that such screw pumps with appropriate diameter and screw pitch can be used for water desalination. The study provides an insight into the design of multifunctional nanodevices for not only water transport but water desalination in practical applications.

Unidirectional water flow at the nanoscale is of significant importance because of its broad applications in a variety of fields such as nanofiltration^1^,^2^,^3^,^4^, desalination of seawater^5^,^6^,^7^ and drug delivery^8^,^9^. In order to generate continuous water flow, unbalanced external field usually has to be employed, such as hydrostatic pressure^10^, osmotic imbalance^11^,^12^, electric fields^13^,^14^,^15^,^16^,^17^,^18^, and thermal gradient^19^,^20^. As a fundamental mechanical motion, rotation also exhibits excellent pumping ability when properly used^21^. The asymmetry of transport system must be satisfied when rotation is used to drive unidirectional water flow^22^. Recently, researchers have found that the rotating chiral carbon nanotube (CNT) could work as a nano pump to drive water without the help from external devices^22^. The coupling of rotation and chiral vacancy modified CNT is also used in a nanofluidic desalination device to turn salt water into fresh water^23^.

With the development of nanotechnology, significant process has been made towards the fabrication of nanostructures with functionalized morphologies^24^,^25^,^26^. Helical nanowires have attracted increasing attention due to its unique asymmetric geometry^27^,^28^. Various materials, such as metals, zinc oxide (ZnO) and polymers are frequently adopted to produce helical nanowires^27^,^28^,^29^,^30^,^31^,^32^. Helical nanowires with four kinds of cross section shapes have been frequently investigated, which are circular, elliptical and rectangular. For the helical nanowires with elliptical cross sections, they can be classified as normal or binormal depending on the orientation of the ellipse^33^,^34^. For the most typical nanowires with circular cross section, several fabrication methods have been reported in the experiments, such as glancing angle deposition (GLAD)^35^, chemical vapor deposition (CVD)^25^,^36^,^37^,^38^,^39^ and template electrosynthesis^27^,^28^. A top-down 3D direct laser direct writing (DLW) can even realize the mass production of the helical nanowires^40^. Researchers have also pointed out that helical nanowires of almost arbitrary shape can be designed and fabricated using this method^40^. As a typical example of normal helical nanowires, the ZnO nanowires could be synthesized via thermal evaporation, with the sequence of six straight blocks, each block growing in a given crystalline direction^41^. In many synthesis processes, the configuration parameter could be reduced to the nanometer level^28^,^41^. For helical nanowires with rectangular cross section, research reveals that it can also be curved out from bulk material^42^.

In experiments, the rotating helical nanowires usually serve as nanoswimmers, which can be propelled wirelessly in fluidic environments with good control^27^,^35^, or even further assembled into more complex micro/nanodevices and systems^25^,^39^,^43^. Magnetic fields are commonly used to realize the rotation of helical nanowires^27^,^28^,^35^,^40^,^44^,^45^. Besides, the rotation of small molecules has also been proven feasible by optical, electrical and chemical means^46^,^47^,^48^,^49^. Here in our work, the rotating helical nanowires are used to pump water flow for the first time. When embedded inside any channels, the rotation of the nanowires can offer the water molecules an efficient translation along the helical axis. In this sense, a nano screw pump can be designed with the rotating helical nanowires and a nanochannel. The design concept of the proposed nano screw pump is illustrated in Fig. 1. A water molecule was transported directed in a helical fashion, as shown by the dotted trajectory line. From the perspective of practical applications, it is essential to discuss the feasibility of proposed pump from the following two aspects. Firstly, it has been verified that helical nanowires with the aforementioned four kinds of cross section shapes can be synthesized experimentally. However, when used for water transport, it is essential that the nanowire surface perpendicular to the helical axis roomy enough to accommodate more water molecules during the transport process. Therefore, binormal helical nanowires are not fit for pumping unidirectional water flow. Helical nanowires of circular cross section with large wire radius, normal helical wires, and helical nanowires with rectangular cross section will all make excellent candidates. Secondly, it should be noted that any nanochannel would work here as long as the helical nanowire could be inserted and rotated. As early as in 2002, researchers have synthesized helical crystalline silicon carbide nanowires covered with a silicon oxide sheath based on the screw-dislocation-induced growth mechanism in experiments^43^. The system scale is restricted within dozens of nanometers, which proves the feasibility of our nano screw pump in reality. Besides, the encapsulations of nanowires inside graphene scrolls or CNTs have also been proved feasible^50^,^51^. However, because the helical nanowire serves as the core component of the screw pump and exhibits pumping capacity, details of the nanochannel do not matter here, which provide more possibilities for the channel selection. The combination of helical nanowire and arbitrary nanochannel will make excellent nano screw pump. Due to the accessibility of helical nanowire and nanochannel, the nano screw pump can be easily realized in experiment and scaled up for practical use. As for the scale-up of this pump, one point that needs special care is that when the screw pump is scaled to the certain extent when the gravity cannot be neglected, the nanochannel must be connected to the nanowire as seamlessly as possible to avoid the liquid leakage and increase water flux.

In the present work, we intend to provide a comprehensive assessment on the pumping capability of such nano screw pump and investigate the transport behavior of the water flow inside. With the help of molecular dynamics (MD) simulations, the dependence of water flux on blade’s surface energy and screw’s diameter were systematically investigated using a simplified screw pump model. Moreover, three modes of water transport were observed inside nano screw pumps: cluster-by-cluster, pseudo-continuous and linear-continuous. The first two nonlinear transport modes are jointly determined by weak hydrogen bonding due to space confinement and hydrophobic blades. Despite the discontinuous transport, the cluster-by-cluster mode still guarantees relatively efficient transport, as the constraint of hydrogen bonds is negligible. As a novel-designed nanodevice, our screw pumps with appropriate diameter and screw pitch can also be used for separation of salt ions, which may provide a new clue for water desalination in future applications.

## Methods: Modeling and Simulation

**Figure 1:**
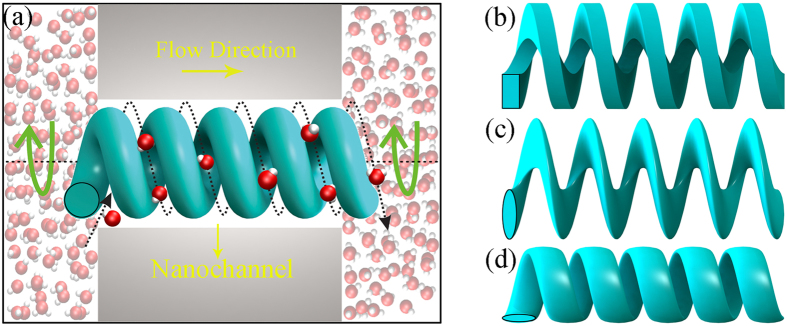
Design concept of the nano screw pump. (**a**) By virtue of the rotating helical nanowire with circular cross section embedded inside a nanochannel, water molecules are transported directed in a helical fashion. The trajectory of a single water molecule is illustrated by the dotted line. (**b**) Helical nanowires with rectangular cross section. (**c**) Normal helical nanowire. (**d**) Binormal helical nanowire.

**Figure 2:**
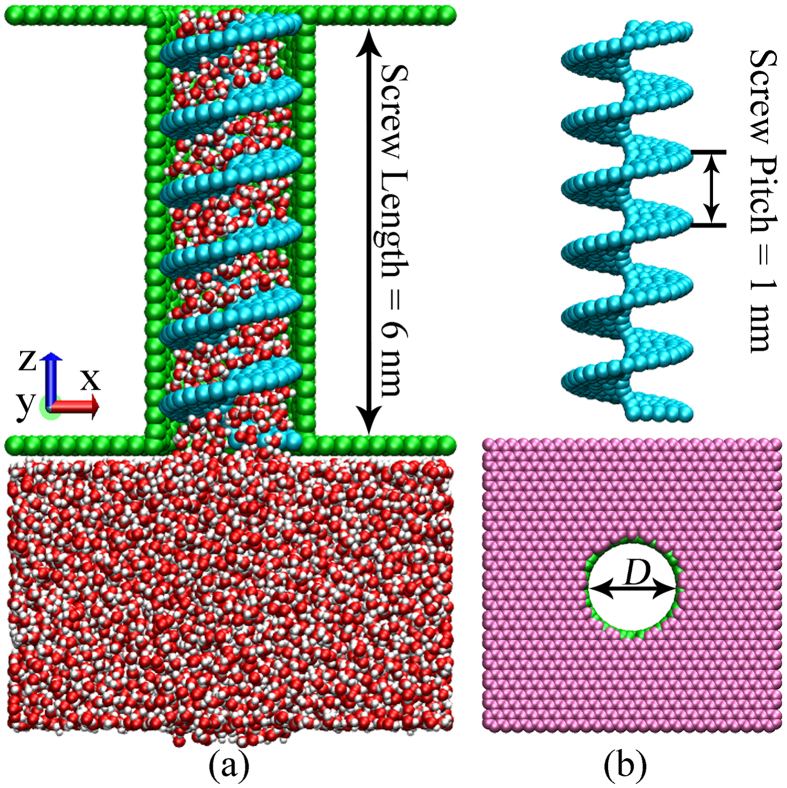
(**a**) Snapshot of the nano screw pump system. An uncapped CNT with a helical blade inside is embedded between two parallel graphene sheets. Water molecules are transported upwards from the lower reservoir. The screw is 6 nm in length and screw pitch is 1 nm. Note that part of carbon nanotube is not displayed for clarity. (**b**) Side view of blades (top) and vertical view of graphene sheets and CNT (bottom). Three CNT diameters (D) are used: 1.36 nm, 1.90 nm and 2.18 nm.

To observe the water transport mechanism as clearly as possible, a simplified nano screw pump is adopted in the MD simulations, as shown in Fig. 2(a). An uncapped CNT with a helical blade inside was embedded along the z direction in two graphene sheets. In order to increase the computational efficiency, a minimal number of atoms are adopted to model the helical blade by preserving the crucial structural feature of helical nanowires and CNT is adopted as nanochannel. As shown in the bottom panel of Fig. 1(b), appropriate openings were made in the graphene sheet allowing water to pass through the nanotube. The positions of all carbon atoms in the graphene and CNT were held fixed during the simulations. The asymmetric helical structure leaves the blade able to rotate only in a specific direction in order to drive the water flow. In the current work, the blade was rotated clockwise as viewed from above. The screw pump was 6.0 nm in length and the screw pitch was 1.0 nm. Three diameters were used: 1.36 nm, 1.90 nm and 2.18 nm for (10, 10), (14, 14) and (16, 16) CNTs, respectively. The corresponding blade diameters were 1.0 nm, 1.5 nm and 1.8 nm. In the nanoscale, since gravity is not considered, water molecules will always fill the nanochannel due to the blade attraction and water flux will be enhanced with the increasing channel diameters. Thus, the seemingly seamless bond of blade and CNT in our MD simulations is only to avoid making the nanochannel diameter as another variable to determine the water flux, which is not the focus of our work. It can be imagined that the water flux will be greatly increased with larger nanochannels in practical applications. The density of the water was fixed at 1 g/ml.

Water molecules in these MD simulations were modeled with the SPC/E model^52^, which includes a Lennard-Jones (LJ) term and a Coulombic term. The long-range Coulombic interaction was calculated using a particle-particle particle-mesh (PPPM) solver^53^ with an accuracy of 10^−4^. The solid-liquid interactions between the water molecules and blades are described using the LJ interactions


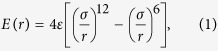


where *r* is the distance between any pair of atoms, and *ε* and *σ* are the depth of potential wells and the effective molecular diameter, respectively. The parameters for solid-liquid interactions were chosen for *σ*_*ls*_ = 3.0 Å. *ε*_*ls*_ is varied from 0.20 to 0.40 kcal/mol in subsequent simulations to explore the effects of blade wettability on water transport. In this work, the carbon-oxygen LJ potential was adopted from a previous work with the parameters *σ*_*CO*_ *=* 3.19 Å and *ε*_*CO*_ = 0.0937 kcal/mol^54^. The cutoff distance for these LJ interactions is set to 10.0 Å.

These simulations were performed with the LAMMPS package^55^. Simulations were carried out in the constant-volume and constant-temperature (NVT) ensemble. The size of simulation box is 3.2 nm × 3.2 nm × 9.4 nm while the system temperature is kept at 300 K using the Nose-Hoover thermostat. Periodic boundary conditions are employed in all directions. The equations of motion were integrated using a velocity-Verlet scheme with a time step of 1.0 fs. We conduct 50 ns MD runs for each simulation system, and the last 40 ns of the trajectories are used for further analysis. Visual molecular dynamics (VMD) is used for the visualization of water transport inside the screw pump^56^.

## Results and Discussion

### Determination of blade’s rotating frequency

**Figure 3:**
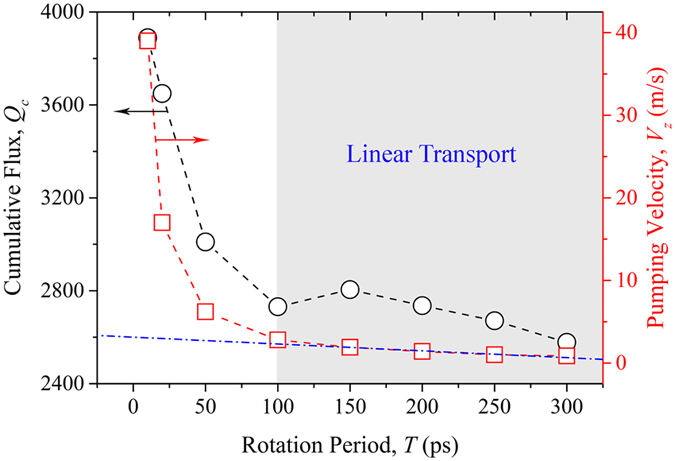
Cumulative flux Q_c_ and pumping velocity V_z_ with T ranging from 10 ps to 300 ps in screw pump. The diameter is 2.18 nm. For each rotation frequency, the blade is rotated for 200 circles. Q_c_ and V_z_ are demonstrated by the circle and square, respectively. The pumping velocity varies linearly within the gray area. The points are connected to guide the eye. The dash dot line helps to illustrate the linear change of V_z_.

When the blades rotate at a low frequency, few or even no water molecules are transported for lack of sufficient driving force. However, when the blade rotates quickly, even though the interactions between the blades and water molecules provide extra thrust for water entrance, water flux is still poor as the interaction is not large enough to balance the effects of impeded water entrance. Thus, selection of a proper rotation period (*T*) is of vital importance for efficient droplet transportation. In these simulations, a series of *T* values were tested on the *D* = 2.18 nm screw pump using Ɛ_*sl*_ = 0.3 kcal/mol. The blades were rotated for 200 circles at different rotation frequencies. The cumulative flux (*Q*_*c*_) and pumping velocity of water molecules (*V*_*z*_) are demonstrated in Fig. 3. It should be noted that *Q*_*c*_ is defined as the total number of water molecules that have crossed one end of the screw system which had previously entered through the other end. With the decay of T, more water molecules are transported after the same 200 circles. As *T* decreases from 100 ps to 10 ps, *Q*_*c*_ increases markedly. However, when *T* varies from 100 ps to 300 ps, *Q*_*c*_ does not change significantly but decreases only slightly according to the overall trend. From the perspective of pumping velocity, it exhibits linear decrease with the increase of *T* when *T* > 100 ps, but increases dramatically as *T* decreases. A ‘linear transport’ region can be defined, as shown by the gray area in Fig. 3. The nonlinear transportation with extremely high water flux at the left panel may be related to the inertia effect arising from the high rotation frequency, which is not the focus of our work. In order to both make sure the linear transport and reduce computational burden, the critical value of 100 ps was selected as rotation period in our simulations.

### Wettability influence on water transport

Here the *D* = 2.18 nm screw pump served as an example to investigate the wettability influence on the water transportation. Consistent with a previous study^57^, the contact angles for *ε*_*ls*_ range from 0.20 to 0.40 kcal/mol in 0.05 kcal/mol increments were 109°, 101°, 80°, 69°, and 50°, respectively.

**Figure 4:**
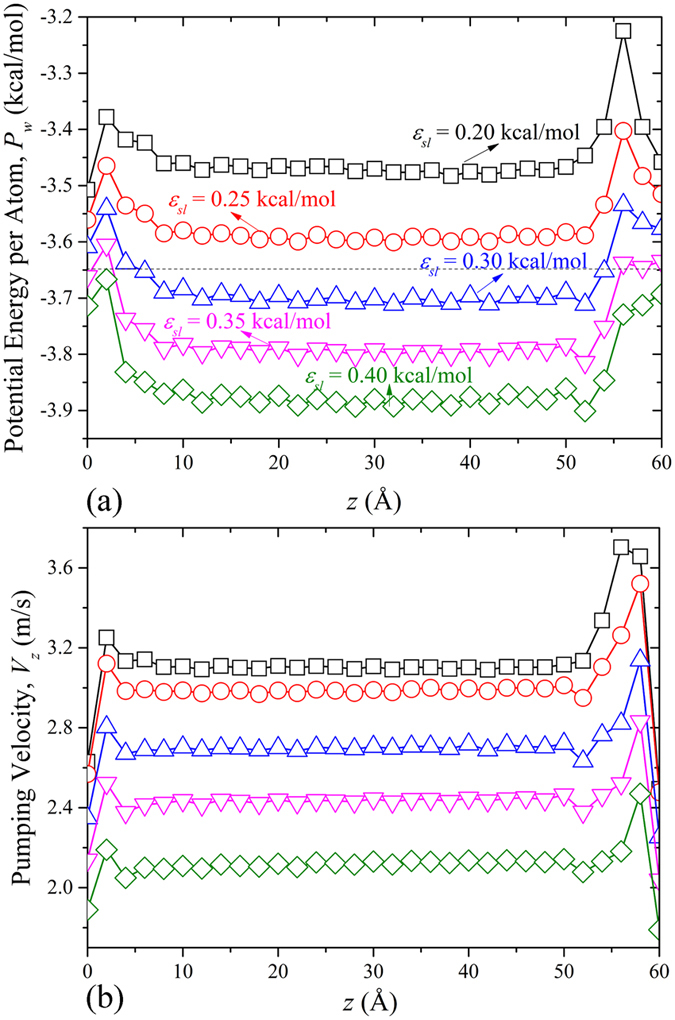
(**a**) Potential energy per atom in water molecules (P_w_) inside D = 2.18 nm screw pump along the z-axis with ε_ls_ ranging from 0.20 to 0.40 kcal/mol. (**b**) Corresponding water pumping velocity V_z_ along the z-axis.

Figure 4(a) shows the profiles of potential energy per atom of water molecules (*P*_*w*_) inside CNTs along the z-axis. It is observed that there is a potential barrier when water enters or escapes from the CNT. However, in the middle of the CNT, the potential energy of water molecules tends to be flat, indicating that the water molecule distribution is homogeneous. As shown, the more hydrophilic the blade is, the lower *P*_*w*_ becomes. The dotted line denotes the potential energy of bulk water, which served as a reference here. For the two screw pumps with hydrophobic blades, the potential energy of water molecules inside the screw was even higher than that of bulk water, which indicates that the water molecules adsorbed on the blade tended to be more unstable. The energy barrier at the entry decreased as *ε*_*ls*_ increased, as more hydrophilic blades provide larger attraction for water molecules to enter the screw pump. While for the exit end, the extremely high energy barrier in the hydrophobic cases indicates that it is more energetically favorable for water molecules to be thrown in to the upper reservoir directly, rather than adsorbing on the blades surface. When the blade attraction is large enough to overcome the centrifugal force in the hydrophilic cases, the water molecules transported to the exit can still stuck on the blade surface and energy barrier decays accordingly. The water behavior at the exit end can also be verified by the pumping velocity in the last pitch, as demonstrated in Fig. 4(b). For different levels of blade wettability, the pumping velocity increased with decreasing solid-liquid interactions due to weaker resistance arising from blades adsorption. The increase in velocity at both ends confirms the existence of energy barrier, while the constant water velocity in the middle region indicates that the water transport is steady.

**Figure 5:**
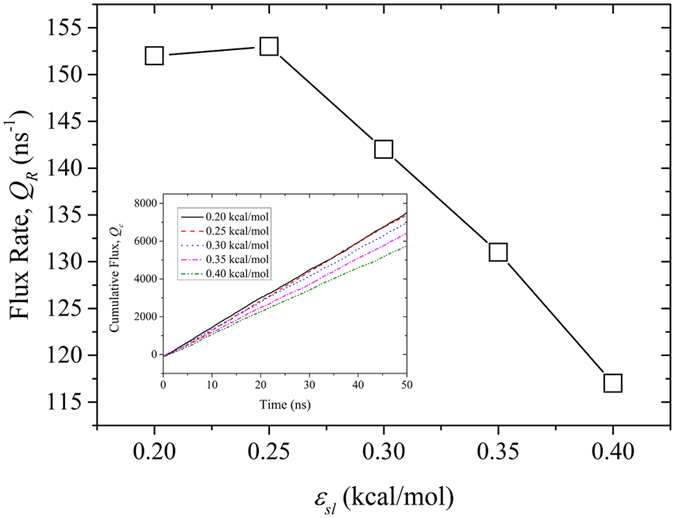
Flux rate (Q_R_) inside *D* = 2.18 nm screw pump as a function of the blade wettability. The points are connected to guide the eye. The inset shows cumulative flux (Q_c_) as a function of time for different levels of blade wettability.

Figure 5 shows the line of flux rate (*Q*_*R*_) as a function of blade wettability in the *D* = 2.18 nm screw pump. *Q*_*R*_, which is defined as the total number of water molecules per nanosecond that have crossed one end of the screw that had previously entered through the other end, decreases with increasing blade wettability on the whole. *Q*_*R*_ can reach up to 153 ns^−1^ when *ε*_*ls*_ = 0.25 kcal/mol. As illustrated in the inset, *Q*_*c*_ is basically inversely proportional to *ε*_*ls*_ after 50 ns. Linear water transport is observed as the result of constant pumping velocity. Thus, *Q*_*c*_ can be expressed as follows:


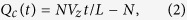


where *L* is the length of the screw pump.

The last 40 ns of the simulation were used for analysis, so Q_R_ was as follows:


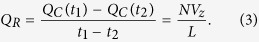


Here t_1_ = 50 ns, and t_2_ = 10 ns.

The number of water molecules inside the screw pump and the pumping velocity are here found to jointly dominate the water flux. It can be observed from Fig. 4 that the pumping velocity exhibits monotonic increase with the decrease of blade wettability. However, the flux rate slightly falls off when *ε*_*ls*_ is 0.2 kcal/mol. Through checking the number of water molecules inside the screw pump, we can conclude that it is the fewer water molecules arising from loose arrangement on the hydrophobic blade that caused the discrepancy.

**Figure 6:**
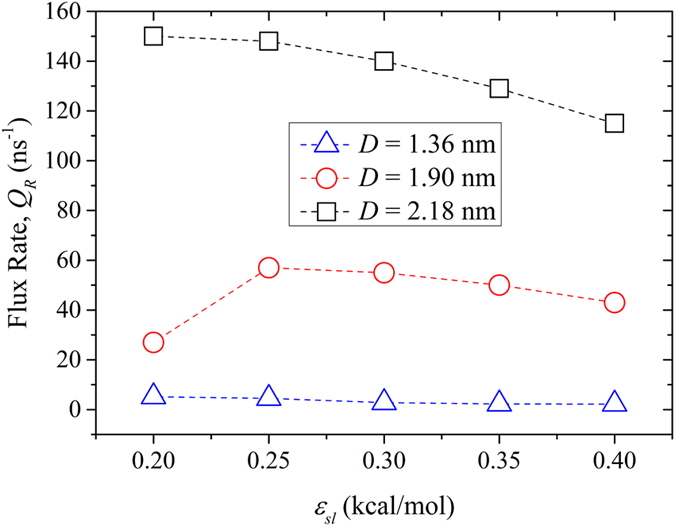
Flux rate (Q_R_) as a function of the blade wettability for screw pumps with different diameters.

The flux rate inside the screw pumps with three different diameters is shown in Fig. 6. Larger screw’s diameter corresponded to higher water flux. The flux rate decreased markedly with the decrease in screw’s diameter. The high flux rate in the *D* = 2.18 nm screw pump can be attributed to the bulk water configuration at larger diameters. For the smallest screw pump, *Q*_*R*_ was merely several molecules per nanosecond. In accordance with the *D* = 2.18 nm screw pump, *Q*_*R*_ decreased with the increasing wettability in principle. There was an evident drop in water flux rate for the *D* = 1.90 nm and *ε*_*ls*_ = 0.2 kcal/mol screw pump. This discrepancy can be attributed to the ordered structures that water took on due to the confined space. This will be discussed in detail in the following subsection.

### Diameter-dependent water structures inside screw pumps

**Figure 7:**
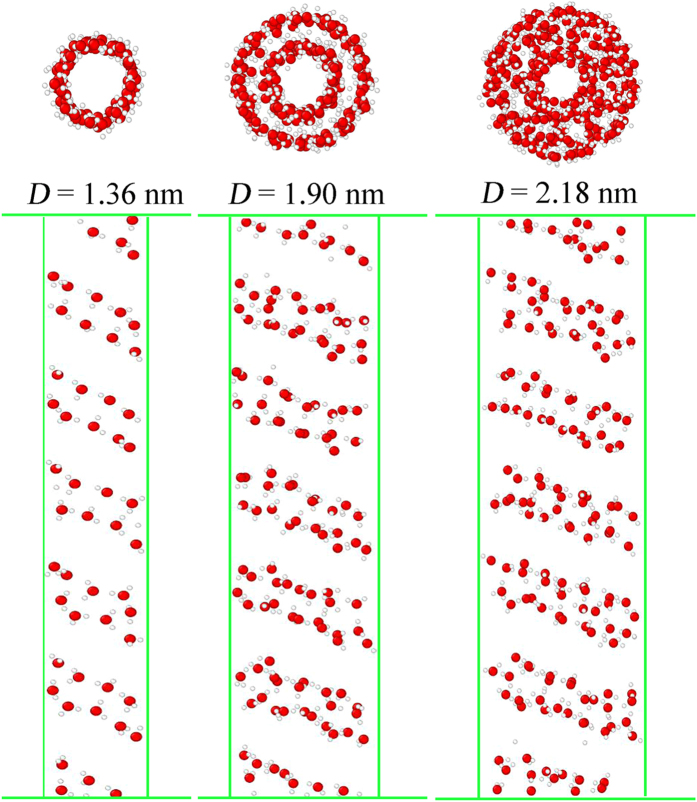
(Top panel) Top view of water structures inside the screw pumps with D = 1.36, 1.90 and 2.18 nm. (Bottom panel) Corresponding water structures inside the back half tube from side view. Green lines denote outlines for CNT and graphene.

To investigate the influence of screw’s diameters, snapshots were taken of water structures in the screw pumps (Fig. 7). In *D* = 1.36 and 1.90 nm screw pumps, the water molecules formed ordered single and double ring structures in the radial direction. The water molecules belonging to two different chains were connected by hydrogen bonds. With larger screw’s diameter (*D* = 2.18 nm), the water molecules were concentrated closely in a disorderly manner, like the bulk water. Moreover, as viewed from the side, water molecules formed double-file chain in the former two cases. Especially for *D* = 1.36 nm case, the double-file chain is fairly regular in every pitch due to space confinement. For the *D* = 2.18 screw pump, the disordered molecule arrangement in the radial direction inevitably results in irregular molecule distribution in the z direction. In this way, the water molecules form ordered structures in the former smaller screw pumps, but more inclined to bulk water in the *D* = 2.18 screw pump, which ensures that the water transport is linear and continuous.

**Figure 8:**
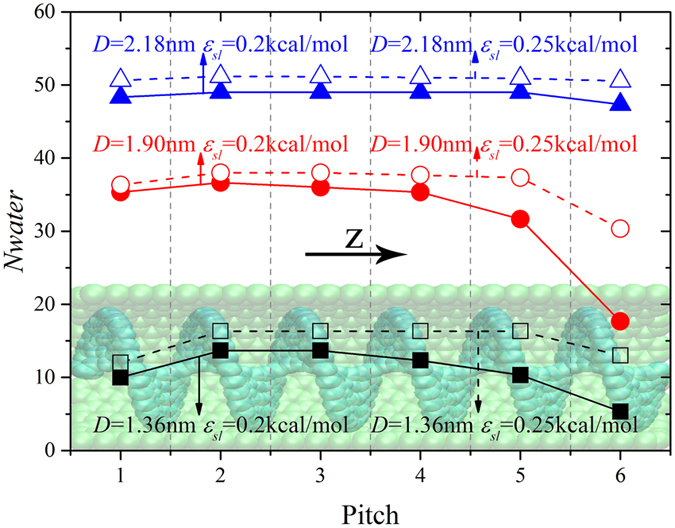
Number of water molecules in each screw pitch (Nwater) along the z direction. For D = 1.36, 1.90 and 2.18 nm, the results are shown by square, circle and triangle. For each screw’s diameter, results are provided when ε_ls_ = 0.2 kcal/mol and ε_ls_ = 0.25 kcal/mol, which are denoted by solid and hollow symbols, respectively. The points are connected to guide the eye. The inset shows the schematic of corresponding pitch along the z direction.

In order to determine the reason for flux rate decline in the *D* = 1.90 nm hydrophobic screw pump, we have calculated corresponding number of water molecules in each pitch (*Nwater*) during the transport process in Fig. 8. For comparison, extra results when *ε*_*ls*_ = 0.25 kcal/mol are also provided. The number of water molecules declines with decreasing screw’s diameter. Considering the *D* = 2.18 nm screw pump, the number of water molecules exhibits no remarkable difference in any pitch. This indicates that the water molecules entering the screw pump are balanced with ones exiting from it. In this way, the water transport inside the screw pump is continuous. Even in the most hydrophobic case, bulk water was able to maintain the continuous transport. When smaller screw’s diameters were considered in the other two cases, the water molecules were basically homogeneous in the middle region at *ε*_*ls*_ = 0.25 kcal/mol. The decline at the input end can be attributed to the impeded water entrance by blade rotation when the blades were hydrophobic. When transported to the top pitch, the water molecules tended to be thrown out of the screw pump instead of adsorbing on the hydrophobic blades, which is responsible for the few water molecules at the top end. However, as the blades became more hydrophobic, results clearly showed that the number of water molecules started to exhibit a monotonic decrease from the second pitch along the z direction, which indicates that the water transport was no longer continuous. It should be noted that there were small differences between the two smaller screw pumps. For the *D* = 1.90 nm case, there was a sharp decline in the number of water molecules in the last pitch. For the smallest pump, the number of water molecules decreased continuously in the pitches along the z direction. Considering the completely opposite trend in the flux rate in the two smaller screw pumps when *ε*_*ls*_ = 0.2 kcal/mol (Fig. 6), it is reasonable to conclude that there are distinct transport modes in the two smaller hydrophobic screw pumps.

### Three modes of water transport inside nano screw pumps

**Figure 9:**
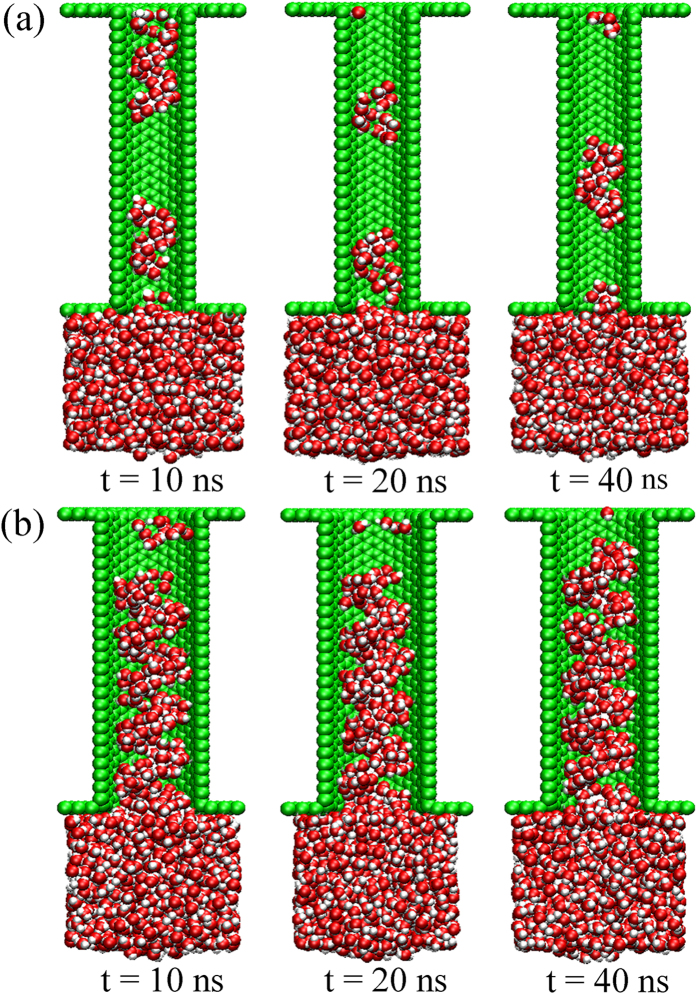
Sequence of MD snapshots of water transport inside (**a**) D = 1.36 nm and (**b**) D = 1.90 nm screw pumps. The solid-liquid interaction used here was 0.2 kcal/mol. Note that blade and part of CNT are not displayed for clarity.

Figure 9 shows the MD results of water transport inside the *D* = 1.36 nm and *D* = 1.90 nm hydrophobic (*ε*_*ls*_ = 0.2 kcal/mol) screw pumps. In the smallest pump, each water molecule was connected to the adjacent one by single hydrogen bonds in the radial direction, which left these single-file chains very fragile. The high energy state of the molecules also undermined the stability of ordered water structures. As a result, these chains of water molecules broke up during transport. Once the breakage of hydrophobic bonds occurred, water molecules clusters can gain higher velocity on the hydrophobic blade for lack of resistance. Thus, the water flux superiority in the smallest hydrophobic screw pump could still be guaranteed. Similarly, in many previous works, fast transport of water through nanochannels can be achieved by reducing the number of hydrogen bonds, such as breaking hydrogen bonds using electric resonance^16^, or restricting hydrogen bonds formation in narrow channels^58^. The situation is different when screw’s diameter is slightly larger (*D* = 1.90 nm). As shown in Fig. 9(b), the water molecules were bonded tightly to each other due to the double chain files in both the radial and axial directions. Thus, it was not possible for the water chain files to break into many clusters during blade rotation. However, when transported to the last one or two pitches, the water molecules tend to escape from the rotating hydrophobic blades instead of adsorbing onto it due to the weak solid-liquid attractions. The sharp decline in the number of water molecules in the last two pitches shown in Fig. 8 also confirmed this. Because it is energetically costly to rupture the whole water chain, the transport velocity decreased considerably, which results in low flux rates. In a word, the nonlinear water transport tends to occur in the cases when the blade is too hydrophobic or the space is confined, such as small screw’s diameter or small pitch.

Based on all these discussions, we find that three modes can be identified for the water transport in nano screw pumps.

1. “Cluster-by-cluster”. This is best represented by *D* = 1.36 nm and *ε*_*ls*_ = 0.2 kcal/mol screw pump in our work. In this mode, the water molecules assembled into small irregular clusters that moved together through the screw, and one cluster followed the next. Despite the discontinuity, the rapid water transport could still be guaranteed because of negligible hydrogen bond resistance.

2. “Pseudo-continuous”. One typical example is the *D* = 1.90 nm and *ε*_*ls*_ = 0.2 kcal/mol screw pump. The continuous water chain seems to be preserved. However, this was not true for all the water molecules inside the screw pump, as the ones at the top had to break from the chain file in order to escape the screw pump. Under these circumstances, the water flux rate tended to decline as a result of energetically costly cracking from the whole chain file.

3. “Linear-continuous”. This was observed with the *D* = 2.18 nm screw pumps and smaller screw pumps with more hydrophilic blades. The linearly continuous transport can be considered ideal, and high water flux can be attained.

Apart from water transport, we find that the nano screw pump can also be used for ions separation. By creating confined space with appropriate diameter and screw pitch, salt ions can be blocked out when flowing through nano screw pump. In this way, even small organic molecules miscible with water can also be separated out based on different hydrate size. The use of nano screw pump for efficient water desalination will be provided in a separated paper, which may provide a new clue for water desalination in future applications.

## Conclusions

A nano screw pump with helical nanowire embedded into a nanochannel was designed and its water transport mechanism was investigated using MD simulations. By considering different circumstances, three modes of water transport through screw pumps were observed: cluster-by-cluster, pseudo-continuous, and linear-continuous. In most cases, linear water transport could be achieved through blade rotation. The first two modes of transport explained the variation in flux rate between small hydrophobic screw pumps. Through investigation of the influence of blade’s surface energy, the water flux rate was found to exhibit a largely monotonic decrease as blade wettability increased. According to the theoretical predictions, the water flux rate was jointly determined by the number of water molecules inside the screw pump and the pumping water velocity. In this way, transportation was rendered somewhat less efficient due to loosely packed water molecules on hydrophobic blades. As for the influence of screw’s diameter, ordered water chain structures and bulk water dominated in small and large screw pumps, respectively. The weak hydrogen bonding due to space confinement in small hydrophobic screw pumps contributed to the nonlinear water transport. In addition to water transport, it is proposed the application field of nano screw pump could also be extended to water desalination, or even for more general liquid separation.

## Additional Information

**How to cite this article:** Wang, L.Y. *et al*. Design of Nano Screw Pump for Water Transport and its Mechanisms. *Sci. Rep.* **7**, 41717; doi: 10.1038/srep41717 (2017).

**Publisher's note:** Springer Nature remains neutral with regard to jurisdictional claims in published maps and institutional affiliations.
